# Event-Related Potentials Reveal Altered Executive Control Activity in Healthy Elderly With Subjective Memory Complaints

**DOI:** 10.3389/fnhum.2018.00445

**Published:** 2018-11-14

**Authors:** Jesús Cespón, Santiago Galdo-Álvarez, Fernando Díaz

**Affiliations:** ^1^Basque Centre on Cognition, Brain and Language, Donostia, Spain; ^2^Department of Clinical Psychology and Psychobiology, University of Santiago de Compostela, Santiago de Compostela, Spain

**Keywords:** executive functions, simon task, event-related potentials, aging, subjective memory complaints

## Abstract

Several studies reported that healthy elderly with subjective memory complaints (SMC) evolve to Alzheimer’s disease (AD) more frequently than elderly without subjective memory decline. In the present study, we investigated event-related potentials (ERPs) associated to executive control processes taking place during the performance of a Simon task with two irrelevant dimensions (stimulus position and direction pointed by an arrow) in healthy elderly divided in low and high SMC (LSMC, HSMC) groups. P300 was studied as a correlate of working memory. Medial frontal negativity (MFN) was studied as a correlate of conflict monitoring. Whereas the LSMC group showed interference from the stimulus position, participants with HSMC showed interference from both irrelevant dimensions. P300 latency was longer and P300 amplitude was lower when the stimulus position was incompatible with the required response but differences between both groups were not observed. MFN was not modulated in the LSMC group; however, the HSMC group showed larger MFN when the stimulus position and/or the direction pointed by the arrow were incompatible with the required response. These results suggest that participants with HSMC deployed greater conflict monitoring activity to maintain the performance when the target stimulus contained conflictive spatial information.

## Introduction

A substantial number of studies reported that healthy elderly with subjective memory complaints (SMC) are at a higher risk of evolving to mild cognitive impairment (MCI) and Alzheimer’s disease (AD) than healthy elderly without SMC (Dickerson et al., 2007; Glodzik-Sobanska et al., 2007; Jessen et al., 2010; Stewart et al., 2011; Rönnlund et al., 2015). Also, several studies showed that healthy elderly with SMC exhibit greater temporal (Tepest et al., 2008; Striepens et al., 2010; Schultz et al., 2015) and frontal (Toledo et al., 2015) atrophy as well as a higher prevalence of pathophysiological markers related to AD (Visser et al., 2009). Therefore, studying SMC may represent an opportunity for early identifying those individuals who will probably evolve to AD.

The physiological aging is accompanied by a substantial decline of executive functions (Grady, 2012), which is severely aggravated by pathophysiological processes related to AD (Kirova et al., 2015). The term executive functions includes a set of cognitive processes such as working memory, cognitive flexibility, attentional and cognitive control, which are used in our daily life activities to monitor behaviors and implement goal-directed actions (Chan et al., 2008; Diamond, 2013). Some studies reported that healthy elderly with SMC exhibit poorer executive functioning compared to elderly without memory complaints (Steinberg et al., 2013; Stenfors et al., 2013). In contrast, other studies did not find differences in executive functions (Striepens et al., 2011) or in other cognitive processes (van der Flier et al., 2004; Jessen et al., 2006; Peter et al., 2014) according to the level of SMC reported by healthy elderly individuals.

Executive functions were frequently studied by using stimulus-response compatibility tasks (Kornblum et al., 1990) such as the Simon task (Lu and Proctor, 1995; Leuthold, 2011). During the Simon task, participants have to respond to a non-spatial feature (e.g., color, shape) of a lateralized stimulus by pressing one of two response buttons that are lateralized in the same spatial arrangement. Although the stimulus position is irrelevant to perform the task, the reaction time (RT) is longer when the side of the required response is spatially incompatible with the stimulus position (incompatible condition) compared to trials that require an ipsilateral response regarding the stimulus position (compatible condition). Several studies reported that the spatial interference observed during the performance of a Simon task increases during the healthy aging (van der Lubbe and Verleger, 2002; Cespón et al., 2013a) and it is additionally increased in individuals diagnosed of MCI (Pereiro et al., 2014; Cespón et al., 2015) and AD (Castel et al., 2007; Tse et al., 2010).

The temporal dynamic of the interference and cognitive control can by studied by means of distributional analysis of the RTs (DA; Ratcliff, 1979; Ridderinkhof, 2002). This type of analysis usually showed that the interference observed during the stimulus-response compatibility tasks decreases at slower RT because greater cognitive control processes are implemented at slow responses (De Jong et al., 1994; Proctor et al., 2011). Interestingly, several studies reported that interference at slower RTs increases with aging, suggesting a greater difficulty to deploy cognitive control mechanisms to inhibit the irrelevant information in elderly persons (Castel et al., 2007; Juncos-Rabadán et al., 2008). Thus, the DA represents a straight tool to study differences in cognitive control abilities.

The event-related brain potentials (ERPs) technique is an appropriate tool to obtain brain correlates of the cognitive processes taking place during the performance of a cognitive task due to its high temporal resolution. In addition, previous studies suggested that ERP modulations are more sensitive than behavioral measures to incipient physiological processes related to pathological aging (Zurrón et al., 2018). In the present study, executive control functions will be investigated in samples of healthy elderly participants divided in low SMC (LSMC) and high SMC (HSMC) groups. Participants will perform a Simon task that will require responding to the color of a lateralized stimulus (which will be a blue or a red arrow) while ignoring two irrelevant dimensions (stimulus position and direction pointed by the arrow). The two irrelevant dimensions will increase the conflict monitoring demands and consequently, the sensitivity of the task to detect group-related differences (Juncos-Rabadán et al., 2008). Medial frontal negativity (MFN) will be investigated as a correlate of brain activity related to monitoring of the conflictive spatial information whereas P300 will be studied as a correlate of working memory. Both ERP (i.e., MFN and P300) are described in the following paragraphs.

MFN is a response-locked ERP that appears around 50–100 ms after emitting the response and achieves its maximum amplitude in fronto-central electrodes. MFN is a correlate of brain activity generated in the anterior cingulate cortex (Yeung et al., 2004; Masaki et al., 2007; Nessler et al., 2007), which was related to conflict monitoring processes (Botvinick et al., 2001). In this regard, the amplitude of MFN correlates with the amount of neural activity allocated to conflict monitoring (Masaki et al., 2012; Watanabe et al., 2016). For instance, Masaki et al. (2012) reported that the amplitude of MFN was larger in a version of the Simon task that required high levels of conflict monitoring compared to an easier version of the Simon task. As far as we know, previous studies did not focus on the possible modulations of MFN in healthy elderly individuals with low vs. high degree of SMC.

P300 is a stimulus-locked ERP waveform that emerges around 300–600 ms after stimulus presentation. In Simon-type tasks, the P300 was related to working memory update of the required stimulus-response mapping—which is also in line with that suggested by Verleger et al. (2005)—and it achieves its maximum amplitude at parietal regions (Leuthold, 2011; Hoppe et al., 2017). Several studies reported longer P300 latency when the stimulus position is incompatible with the required response (Valle-Inclán, 1996; Leuthold, 2011) and in those conditions where the stimulus-response mapping differs from the previous trial (Melara et al., 2008; Hoppe et al., 2017). Importantly, previous studies reported that P300 latency was delayed in healthy elderly with SMC who performed an oddball task (Braverman and Blum, 2003) as well as in healthy elderly participants with SMC who subsequently evolved to AD (Gironell et al., 2005). Nevertheless, previous studies did not focus on the utility of P300 to obtain correlates of neural activity related to SMC in cognitive control tasks.

The main objective of the present study was to investigate whether healthy elderly with a high degree of SMC show increased interference from irrelevant spatial information and/or alterations in specific cognitive processes associated to the studied ERP components (i.e., MFN and P300) in comparison with healthy elderly with a low degree of SMC. Previous studies reported impaired executive functions in healthy elderly with SMC. Thus, we may hypothesize that HSMC elderly group will show greater interference from irrelevant information (i.e., the stimulus position and the direction pointed by the arrow) and stronger brain activity modulations (as revealed by P300 and MFN) associated to the conflictive information than LSMC elderly group.

## Materials and Methods

### Participants

Thirty-four participants (26 women, 8 men) between 52 and 81 years old were divided into two groups: LSMC (*n* = 18; 13 women, 5 men; age range: 52–81), HSMC (*n* = 16; 13 women, 3 men; age range: 51–74). The participants were recruited from the general population and volunteered to take part in the study. This study has been approved by the USC ethics committee and by the Galicia Clinical Research ethics committee and has been performed in accordance with the ethical standards laid down in the 1964 Declaration of Helsinki. All participants gave their informed consent prior to their inclusion in the study. Also, participants received an explanation about the procedures and type of tasks to carry out as well as the purposes of the study. All the used experimental procedures were exempt of risks for the participants. Moreover, rigorous anonymity of all participants taking part in the project was carefully and strictly preserved according to national and EU legislation. All the participants were right-handed, as evaluated by the Edinburgh Handedness Inventory (Oldfield, 1971). All participants had normal or corrected to normal vision and no history of neurological or psychiatric disorders.

Participants were divided in LSMC and HSMC according to the scores obtained in a standardized memory complaints questionnaire (Benedet and Seisdedos, 1996). In detail, the participants who scored equal or below 16 were included in the low memory complaints group whereas the participants who scored above 16 were included in the high memory complaints (mean scores for each group are summarized in Table 1). In addition to a standardized memory complaints questionnaire (Benedet and Seisdedos, 1996), all the participants conducted an exhaustive neuropsychological evaluation to ensure that they performed within normal parameters and memory complaints were not objectively observed in a standardized neuropsychological assessment. In addition, years of education (reported in Table 1) were considered as proxy variable of cognitive reserve in order to avoid differences between groups in this variable, as cognitive reserve is thought to modulate executive functions (Corral et al., 2006; Cabral et al., 2016).

**Table 1:** This table summarizes means and standard deviations for the main socio-demographic data (direct scores are highlighted in bold; z scores are specified below the direct scores for the performed tests) as well as for the neuropsychological tests that were performed by the participants.

	Age	Sex	Education	Vocabulary WAIS	MMSE	Subjective memory complaints*	GDS*
LSMC	**65.1 (9.1)**	**13 w/5 m**	**9.1 (5.4)**	**48.9 (14.9)**	**28.2 (1.2)**	**13.2 (1.8)**	**1.8 (1.6)**
				0 (1)	0.2 (0.4)	−0.8 (0.4)	−0.4 (0.8)
HSMC	**64.6 (7.1)**	**13 w/3 m**	**8.4 (2.8)**	**49.0 (12.8)**	**27.6 (1.6)**	**20.8 (2.0)**	**3.4 (1.8)**
				0 (0.9)	−0.2 (1.1)	0.9 (0.4)	0.4 (0.9)
**California Learning Verbal Test**							
	**Short term free recall**	**Short term clue recall**	**Long term free recall**	**Long term clue recall**			
LSMC	**10.2 (2.8)**	**10.9 (2.6)**	**11.0 (3.0)**	**11.4 (2.6)**			
	0 (1.2)	−0.1 (1)	0 (1.2)	−0.1 (1.1)			
HSMC	**10.3 (1.6)**	**11.4 (2.6)**	**11.2 (2.1)**	**11.8 (2.2)**			
	0 (0.7)	0.1 (1)	0 (0.8)	0.1 (0.9)			
**Cambridge Cognitive Examination**							
	**Orientation**	**Language**	**Attention**	**Praxis**	**Perception**	**Executive functions**
LSMC	**9.6 (0.5)**	**25.7 (2.2)**	**7.5 (1.2)**	**11.1 (1.1)**	**6.3 (1.8)**	**18.4 (5.2)**
	0.9 (0.9)	−0.5 (1.1)	−0.1 (0.9)	0.5 (0.9)	−0.3 (1.1)	0 (1.1)
HSMC	**9.5 (0.5)**	**25.9 (1.7)**	**7.7 (1.4)**	**11.0 (1.0)**	**7.3 (1.2)**	**18.1 (4.4)**
	−0.1 (1.1)	0.5 (0.8)	0.1 (1.1)	−0.5 (1.0)	0.3 (0.7)	0 (0.9)

*Asterisks denote the existence of significant differences (*p* < 0.05) in the independent samples *t*-tests that we carried out between low subjective memory complaints (LSMC) and high SMC (HSMC) groups on each variable. GDS: geriatric depression scale of Yesavage; MMSE: mini mental state examination*.

The neuropsychological evaluation included the Spanish versions of Mini-mental state examination (Folstein et al., 1975), the vocabulary test of Wechsler Adult Intelligence Scale (Wechsler, 1981), the California Verbal Learning Test (CVLT, Delis et al., 1987), which was used to test episodic memory, and the Spanish version of CAMCOGR (Roth et al., 1998), a subscale of CAMDEX-r (Roth et al., 1986) that includes subscales for assessing specific cognitive domains such as executive functions, language and attention. Also, all the participants performed the Spanish version of the Yesavage geriatric depression scale (Yesavage et al., 1983) in order to exclude depression as an explanation for the SMC.

### Experimental Task and Procedure

**Figure 1:**
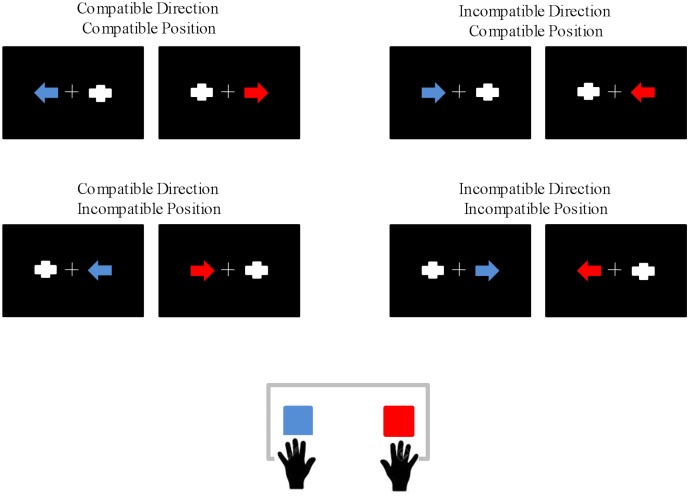
Schematic representation of the eight types of stimuli, which gave rise to the following experimental conditions: Compatible Direction-Compatible Position (CDCP), In CDCP (IDCP), Compatible Direction-Incompatible Position (CDIP), InCDIP (IDIP). Eighty trials per experimental condition were presented, giving rise to 320 trials in total. The response buttons assigned to each color were counterbalanced among participants.

A set of red or blue arrows pointing either left or right were displayed on a screen against a black background. The screen was placed 100 cm in front of the participants. The arrows subtended 2.87° horizontally and 1.72° vertically in the visual field. A gray geometric figure of similar morphology and eccentric position (two orthogonally superimposed bars) was presented in the opposite hemifield to the target stimulus. Importantly, the presence of a contralateral stimulus does not modulate the Simon effect or the main ERP components taking place during Simon-type tasks (Valle-Inclán, 1996; Leuthold, 2011). The arrow and the contralateral non-target stimulus were presented for 125 ms, with an inter-trial interval of 2,000 ms. In order to minimize ocular movements, stimulation was presented within the foveal region, as the external edges of the stimuli were at 5.16° of visual angle with respect to the central cross (Bargh and Chartrand, 2000). Participants were instructed to direct their gaze towards a central cross throughout the task and to respond to the color of a blue or red arrow by pressing one of two horizontally positioned buttons (blue or red) while ignoring the stimulus position and the direction indicated by the arrow (Figure 1).

The two irrelevant dimensions of the task (stimulus position and direction indicated by the arrow) resulted in four experimental conditions according to the compatibility/incompatibility of these irrelevant dimensions with the response to the color: compatible direction-compatible position (CDCP), in CDCP (IDCP), compatible direction-incompatible position (CDIP) and in CDIP (IDIP). After a practice block of 24 trials, a total of 320 trials (80 per condition) were presented in two separated blocks (inter-block break of 90 s). The response hand assigned to each color of the stimulus was counterbalanced among the participants, who were instructed to respond as quickly and accurately as possible.

### EEG Recordings

Forty-seven active electrodes placed in accordance with the 10-10 International System were used for the EEG recordings. The EEG signal was passed through a 0.01–100 Hz analog band-pass filter and was sampled at 500 Hz. The reference electrode was placed on the tip of the nose and the ground electrode at Fpz. Simultaneously to EEG recordings, ocular movement (EOG) recordings were obtained with two electrodes located supra-and infraorbitally to the right eye (VEOG) and another two electrodes at the external canthus of each eye (HEOG). Impedances were maintained below 10 kΩs. Blinks were corrected off-line by use of the algorithm of Gratton et al. (1983).

The signal was passed through a 0.01–30 Hz digital band-pass filter. One-second epochs were extracted: 200 ms pre-stimulus in stimulus-locked ERPs (P300) and 600 ms pre-response in response-locked ERPs (MFN). Epochs with signals exceeding ±100 μV were automatically rejected, and all remaining epochs were inspected individually to identify those still displaying artifacts; the artifact epochs were also excluded from subsequent averaging. Epochs were then corrected to the mean voltage of the baseline (−200 to 0 in stimulus-locked ERPs, −600 to −400 in response-locked ERPs).

### Data Analysis

Similarly to previous studies (e.g., Fischer et al., 2010; Cespón et al., 2013a), trials with incorrect responses or RT outside the 100–1,000 ms range were excluded from the behavioral and the ERP analyses.

The RT, the subtracted interference on each incompatible condition (i.e., IDCP-CDCP, CDIP-CDCP and IDIP-CDCP) and the percentage of errors (PE) were analyzed. To determine whether the magnitude of the interference depended on the speed of response, distributional analysis of the RTs (DA) was carried out (Ratcliff, 1979) for each group (LSMC, HSMC) and type of interference (IDCP, CDIP, IDIP). For this purpose, the RTs were ordered by length, and for each participant, the RTs at the 4 Quintile Intersection Points that divided the distribution into five equal parts (quintiles) were selected.

P300 latency was measured in midline electrodes (Fz, Cz, Pz) as the maximum positive peak detected between 300 ms and 600 ms. P300 was studied at Pz as it is the regions were P300 achieves its maximum amplitude. In addition, considering the posterior to anterior shift of activity related to ageing (Davis et al., 2008), we have also studied P300 at Cz and Fz electrodes in order to investigate possible differences related to group in those scalp locations. P300 amplitude was measured by taking a time window of ±50 ms around the peak latency of each individual participant. Following previous studies (Nessler et al., 2007), MFN was analyzed as the mean amplitude calculated in fronto-central electrodes (AFz, Fz, F3, F4, FCz, FC1, FC2, FC3, FC4) between 60 ms and 110 ms after the RT.

### Statistical Analysis

Independent samples *t*-tests were carried out for age, years of education and the neuropsychological tests performed by participants. Also, Pearson correlation analyses were carried out between years of education, depressive symptoms (GDS), SMC and the performed neuropsychological tests (summarized in Table 1).

RTs and PE were analyzed. The DA also enabled studying whether the temporal dynamic of the interference differed on each group. In detail, we investigated whether interference was significant for each quintile and group separately by applying one sample *t*-tests against 0. Moreover, to study whether interference differed significantly between both groups throughout the DA on each condition, repeated measures ANOVAs were carried out for each interfering condition (i.e., IDCP, CDIP, IDIP) with a between-subject factor, Group (two levels: LSMC, HSMC) and a within-subject factor: Quintile Intersection Point (q) (four levels: q1, q2, q3, q4). Also, in order to determine any differences in RTs, PE and the studied ERP components (P300 and MFN), mixed ANOVAs were carried out with two within-subject factors, Position (two levels: Compatible and Incompatible) and Direction (two levels: Compatible and Incompatible) and one between-subject factor, SMC (two levels: LSMC, HSMC).

The Greenhouse-Geisser ε correction value for the degrees of freedom was used when necessary and the corresponding α levels were determined. For ANOVAs, eta square is provided as a measured of the effect size. When the ANOVAs revealed significant effects due to the factors and their interactions, *post hoc* comparisons of the mean values were carried out by paired multiple comparisons (adjusted to Bonferroni).

## Results

### Neuropsychological Tests and Socio-Demographic Variables

Independent samples *t*-tests carried out on socio-demographic and neuropsychological tests revealed that scores of the geriatric depression scale were greater in high than in low memory complaints group (*t*_(32)_ = 2.68, *p* = 0.011). No differences were obtained in any of the other studied dependent variables.

Pearson correlation analysis between years of education and neuropsychological tests reveal the existence of positive associations between years of education and executive functions of CAMCOG (rxy = 0.605, *p* < 0.001), language of CAMCOG (rxy = 0.611, *p* < 0.001), short-term recall memory of CLVT (rxy = 0.411, *p* = 0.016) and vocabulary test of WAIS (rxy = 0.655, *p* < 0.001). Also, the scores obtained in the depression scale of Yesavage correlated with memory complaints (rxy = 0.490, *p* = 0.003). Neuropsychological scores and socio-demographic variables are summarized in Table 1.

### Simon Task: Behavioral Results

For RTs, the repeated measures ANOVA (Position × Direction × Group) revealed an effect of the Position (*F*_(1, 32)_ = 80.9, *p* < 0.001, $\eta_{\text{p}}^{2}$ = 0.717), as the RT was longer when the stimulus position was spatially incompatible with the required response (*p* < 0.001). For PE, the repeated measures ANOVA (Position × Direction × Group) revealed an effect of the Position (*F*_(1, 32)_ = 17.0, *p* < 0.001, $\eta_{\text{p}}^{2}$ = 0.717). The ANOVA did not reveal an effect of Direction, Group or any significant interaction. RT and PE are recapped in Table 2.

**Table 2:** This table summarizes the means and standard deviations for the obtained behavioral results (Reaction Times, RTs and Percentage of errors, PE) for each group—LSMC and HSMC—and experimental condition: Compatible Direction-Compatible Position (CDCP), Incompatible Direction-Compatible Position (IDCP), Compatible Direction-Incompatible Position (CDIP), Incompatible Direction-Incompatible Position (IDIP).

RT	CDCP	IDCP	CDIP	IDIP
LSMC	555 (88)	557 (77)	593 (80)	596 (79)
HSMC	552 (61)	558 (53)	614 (71)	607 (71)
**PE**	**CDCP**	**IDCP**	**CDIP**	**IDIP**
LSMC	2.8 (3.7)	3.0 (3.1)	6.4 (4.5)	5.4 (4.5)
HSMC	2.5 (3.1)	1.4 (1.6)	5.5 (4.6)	4.0 (3.5)

**Figure 2:**
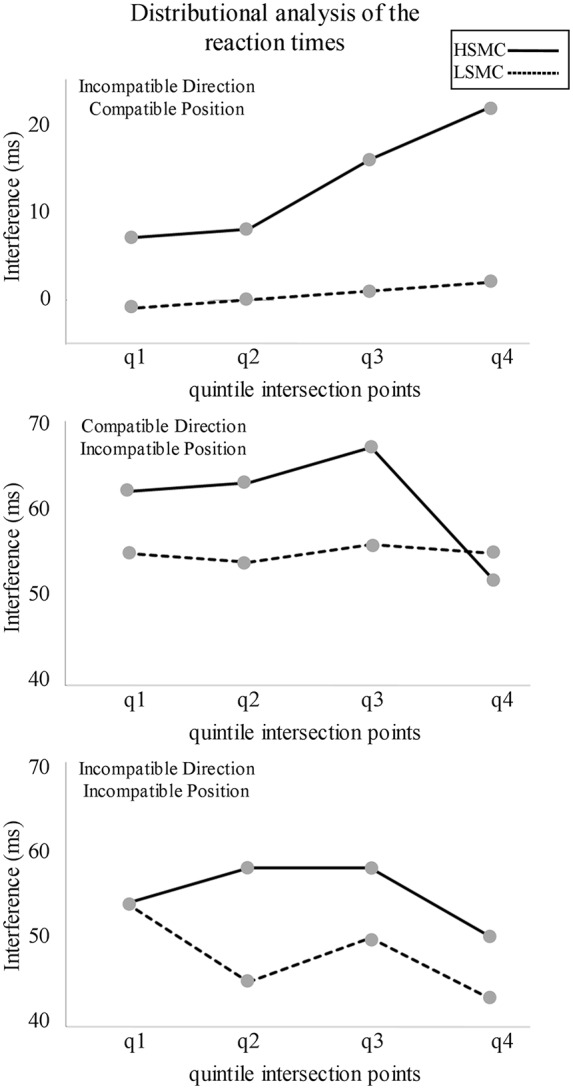
Distributional analyses of the reaction times (RTs) in the three experimental conditions in which a spatial conflict was present. Interference from the spatial position was significant in all the quintiles for both groups. Interference from the direction pointed by the arrow was significant in q3 for the high subjective memory complaints (HSMC) group.

One sample *t*-tests conducted for the DA (which are graphically represented in Figure 2) revealed that interference for LSMC group was significant in CDIP (q1: *t*_(17)_ = 12.6, *p* < 0.001; q2: *t*_(17)_ = 11.1, *p* < 0.001; q3: *t*_(17)_ = 14.5, *p* < 0.001; q4: *t*_(17)_ = 8.7, *p* < 0.001) and IDIP (q1: *t*_(17)_ = 12.7, *p* < 0.001; q2: *t*_(17)_ = 8.2, *p* < 0.001; q3: *t*_(17)_ = 9.7, *p* < 0.001; q4: *t*_(17)_ = 5.9, *p* < 0.001). For the HSMC group, interference was significant in IDCP (q3: *t*_(15)_ = 2.5, *p* = 0.024), CDIP (q1: *t*_(15)_ = 6.8, *p* < 0.001; q2: *t*_(15)_ = 6.8, *p* < 0.001; q3: *t*_(15)_ = 7.2, *p* < 0.001; q4: *t*_(15)_ = 4.8, *p* < 0.001) and IDIP (q1: *t*_(15)_ = 8.0, *p* < 0.001; q2: *t*_(15)_ = 8.2, *p* < 0.001; q3: *t*_(15)_ = 5.9, *p* < 0.001; q4: *t*_(15)_ = 3.9, *p* < 0.001). On the other hand, repeated measures ANOVA (Group × Quintile Intersection Point) carried out for each interfering condition (IDCP, CDIP and IDIP) did not reveal any significant difference on the basis of the experimental group.

### Simon Task: Event-Related Potentials

**Figure 3:**
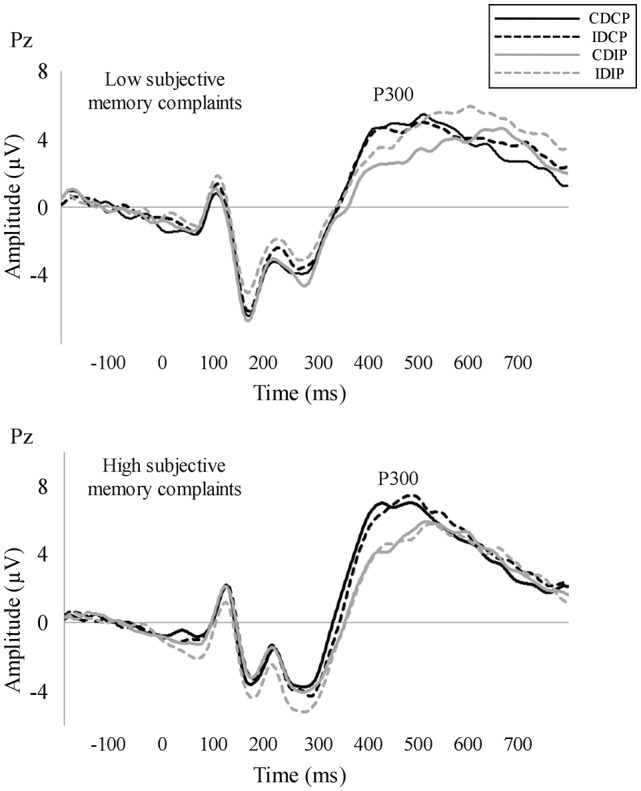
Stimulus-locked averages. P300 was measured as the maximum positive peak observed between 300 ms and 600 ms for each participant and its amplitude in a time window of 100 ms around peak latency. P300 latency was longer and P300 amplitude was smaller in those conditions where the target stimulus position was incompatible with the required response.

For P300 latency, the repeated measures ANOVA (Position × Direction × Group) revealed an effect of Position (*F*_(1, 32)_ = 21.6, *p* < 0.001, $\eta_{\text{p}}^{2}$ = 0.403), as the P300 latency was longer when the stimulus position was incompatible than compatible with the response (*p* < 0.001). For P300 amplitude, the repeated measures ANOVA (Position × Direction × Group) revealed an effect of Position (*F*_(1, 32)_ = 4.2, *p* = 0.048, $\eta_{\text{p}}^{2}$ = 0.117), as the P300 amplitude was lower when the stimulus position was incompatible than compatible with the response (*p* = 0.048). The corresponding repeated measures ANOVA for P300 latency and amplitude did not reveal an effect of Direction, Group or any significant interaction. The grand averages are represented in Figure 3.

**Figure 4:**
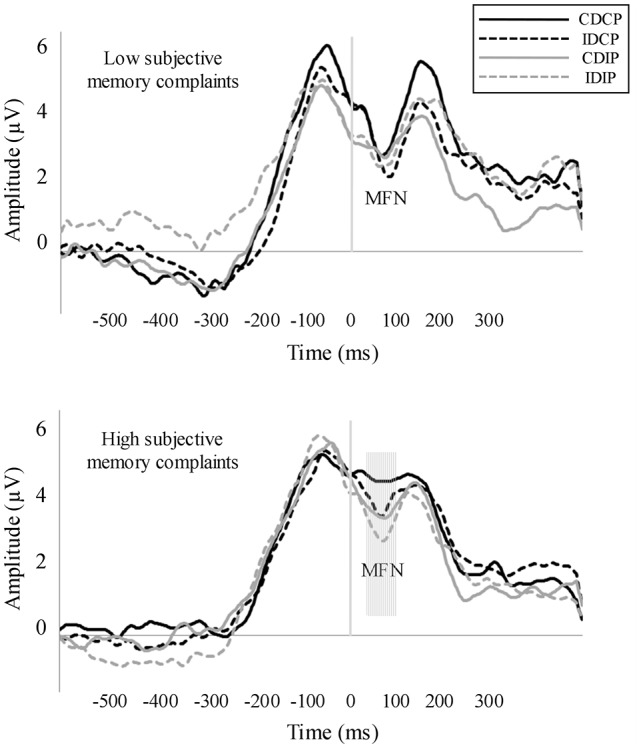
Response-locked averages. Medial Frontal Negativity (MFN) was measured in a pool of fronto-central electrodes. In the HSMC group, MFN amplitude was larger in those conditions where the stimulus position and/or the direction pointed by the arrow were spatially incompatible with the required response.

The repeated measures ANOVA for MFN (Figure 4) revealed a Position × Group interaction effect (*F*_(1, 32)_ = 7.27, *p* = 0.009, $\eta_{\text{p}}^{2}$ = 0.194). For HSMC group, the MFN amplitude was larger when the stimulus was spatially incompatible with the response compared to the trials where stimulus was spatially compatible with the required response (*p* = 0.034). Also, a Direction × Group interaction effect was observed (*F*_(1, 32)_ = 4.39, *p* = 0.044, $\eta_{\text{p}}^{2}$ = 0.121). For HSMC group, the MFN amplitude was larger when the direction pointed by the arrow was spatially incompatible than compatible with the side of the required response (*p* = 0.038). MFN was not modulated in the LSMC group.

## Discussion

The present study investigated neural correlates of executive functions in healthy elderly with high and low SMC (HSMC, LSMC), who performed a Simon task with two irrelevant dimensions (stimulus position and arrow direction). The main results can be summarized as follows: interference from the stimulus position was similar in both groups. Interference from the direction pointed by the arrow was only significant at slow RTs in the HSMC group. The amplitude of MFN was larger when the stimulus position and/or the arrow direction were incompatible with the required response in the HSMC group. MFN was not modulated in the LSMC group. The latency of P300 was longer and its amplitude was lower when the stimulus position was incompatible with the required response for both groups of participants.

The interference effects, as revealed by the overall RT and PE on each experimental condition, did not show differences between both groups of participants. However, the distributional analysis of the RT showed that interference from the direction pointed by the arrow was significant at slow RT in the HSMC (as represented in Figure 2, interference is substantially higher than 0 ms in the quintiles 3 and 4 even if results were not significant in quintile 4 because of the high inter-individual variability) but not in the LSMC group. Interference from the arrow direction at slow RTs, but not at fast RTs, is consistent with previous studies (Pellicano et al., 2009; Proctor et al., 2011; Cespón et al., 2013b) and it was attributed to the required time to process the semantic information (i.e., the arrow direction; Symes et al., 2005; Vainio et al., 2007; Iani et al., 2011). In the present study, stimulus position and arrow direction were combined in the same task. Interference from the stimulus position was greater than interference from the direction pointed by the arrow. These results are consistent with a previous study using a similar Simon task, in which samples of healthy elderly participants showed interference from stimulus position but not from arrow direction (Cespón et al., 2013a). Moreover, these results support that stimulus position attracts attentional resources in a faster manner than the direction pointed by the arrow (Proctor et al., 2011; Cespón et al., 2013b). In general, results from the DA suggest greater difficulty to ignore irrelevant information in the HSMC group and align with previous research that reported impaired executive functions in healthy elderly with SMC (Steinberg et al., 2013; Stenfors et al., 2013).

ERPs allowed establishing differences between HSMC and LSMC groups in brain activity modulations related to the task performance. In detail, the amplitude of MFN was not modulated in the LSMC group. However, individuals with HSMC showed larger MFN in those conditions where the stimulus position and/or the direction pointed by the arrow were incompatible with the required response. These increased MFN amplitudes suggested greater activity allocated to conflict monitoring (Gehring and Willoughby, 2002; Masaki et al., 2007, 2012; Nessler et al., 2007; Watanabe et al., 2016), which could be interpreted as deployment of compensatory mechanisms to maintain an appropriate level of performance (Reuter-Lorenz and Cappell, 2008; Schneider-Garces et al., 2010). In fact, increased MFN was accompanied by a similar degree of interference from the stimulus position in both groups although the direction pointed by the arrow interfered only in the HSMC group. Thus, these results suggest that greater activity related to conflict monitoring in the HSMC group was only partially successful to maintain the performance.

The latency of P300 was longer and its amplitude was lower when the stimulus position was incompatible with the required response, which is consistent with a substantial number of studies using the Simon task (for an electrophysiological review about the Simon task, see Leuthold, 2011). Nevertheless, in contrast to previous studies (Braverman and Blum, 2003; Gironell et al., 2005), P300 did not show differences between HSMC and LSMC groups. These inconsistent results may be attributed to differences between the task paradigm used by previous studies (i.e., an oddball task) and the task paradigm used in the current study (i.e., a Simon task). In detail, in oddball-type tasks (Squires et al., 1975), P300 is a correlate of the comparison between the identity of the target stimulus (which has to be maintained in working memory throughout the task) and the identity of the stimulus that is currently presented (Picton, 1992; Polich, 2007). Instead, in Simon-type tasks, P300 is a correlate of the ability to retrieve or switch the stimulus-response mapping that has been created by the previous trial (Melara et al., 2008; Spapé et al., 2011). In this regard, future studies should directly explore whether persons with SMC exhibit deficits in processes related to working memory update and stimuli comparison rather than in abilities to perform attentional switches.

Some limitations of the present study should be indicated. First, the relatively low sample size and the low differences between both groups in the level of SMC could have precluded the obtaining of stronger differences. Future studies should also applying residual iteration decomposition algorithm to P300 waveforms, as P300 is formed by several subcomponents related to sensorial, motor and sensoriomotor processes (Ouyang et al., 2017; Verleger et al., 2017). Studying these subcomponents separately could lead to observe differences between groups in a specific P300 subcomponent, which remain masked in conventional P300 averages. Also, even if all the participants were free of neurological and psychiatric disorders, memory complaints correlated with depressive symptoms. This result is consistent with the reported relationship between executive deficits and personality traits (Steinberg et al., 2013). Follow up studies would be useful to distinguish memory complaints due to personality traits from memory complaints due to a prodromal neurodegenerative disease. Finally, we should highlight that the mismatch between subjective and objective cognitive functioning may be related to lifestyle factors (e.g., high education level) linked to cognitive reserve, which was associated to improved cognitive functioning (Stern, 2012). Thus, high cognitive reserve persons with SMC may be experiencing a real decline but they would still perform within normal parameters. In fact, according to previous studies (Corral et al., 2006; Lenehan et al., 2015; Cabral et al., 2016), we found correlations between cognitive reserve and cognition (i.e., executive functions, short-term memory and vocabulary). Longitudinal studies considering the relationship between memory or cognitive complaints and cognitive reserve could be useful to set to what extent high levels of cognitive reserve could be masking a real cognitive decline.

In summary, interference from the stimulus position was similar for LSMC and HSMC groups whereas interference from the direction pointed by the arrow was only significant at slow RTs in the HSMC group. The amplitude of MFN—which was related to anterior cingulate cortex activity involved in conflict monitoring—was larger when the stimulus position and/or the direction pointed by the arrow were incompatible with the required response in the HSMC group. Thus, participants with HSMC deployed greater conflict monitoring activity to maintain the performance, which was achieved for the conflict related to spatial position but not for the conflict related to arrow direction. These results suggest the existence of incipient deficits to monitor conflict information in participants with high SMC.

## Author Contributions

JC contributed to experimental design, data acquisition, analyzed the data and wrote the manuscript. SG-Á contributed to experimental design, analyzed the data and critically revised the manuscript. FD contributed to experimental design and critically revised the manuscript.

## Conflict of Interest Statement

The authors declare that the research was conducted in the absence of any commercial or financial relationships that could be construed as a potential conflict of interest.
